# Prospective Validation Obtained in a Similar Group of Patients and with Similar High Throughput Biological Tests Failed to Confirm Signatures for Prediction of Response to Chemotherapy and Survival in Advanced NSCLC: A Prospective Study from the European Lung Cancer Working Party

**DOI:** 10.3389/fonc.2014.00386

**Published:** 2015-01-28

**Authors:** Thierry Berghmans, Lieveke Ameye, Jean-Jacques Lafitte, Benoît Colinet, Alexis Cortot, Ingrid CsToth, Stéphane Holbrechts, Jacques Lecomte, Céline Mascaux, Anne-Pascale Meert, Marianne Paesmans, Michel Richez, Arnaud Scherpereel, Christian Tulippe, Luc Willems, Tiffany Dernies, Nathalie Leclercq, Jean-Paul Sculier, for the European Lung Cancer Working Party

**Affiliations:** ^1^Department of Intensive Care and Oncological Emergencies and Thoracic Oncology Clinic, Institut Jules Bordet, Université Libre de Bruxelles, Brussels, Belgium; ^2^Data Centre, Institut Jules Bordet, Université Libre de Bruxelles (ULB), Brussels, Belgium; ^3^Department of Pulmonary and Thoracic Oncology, Centre Hospitalier Régional Universitaire de Lille, Lille, France; ^4^Department of Pneumology, Grand Hôpital de Charleroi, site Saint-Joseph, Gilly, Belgium; ^5^Department of Oncology, Hôpital Ambroise Paré, Mons, Belgium; ^6^Department of Pneumology, CH Charleroi, Charleroi, Belgium; ^7^Department of Medical Oncology and Hematology, Princess Margaret Cancer Centre, Toronto, ON, Canada; ^8^Department of Pneumology, CHR St Joseph, Warquignies, Belgium; ^9^Department of Pneumology, CH Mouscron, Mouscron, Belgium; ^10^Laboratory of Molecular and Cellular Biology, Université de Liège, Gembloux, Belgium

**Keywords:** non-small cell lung cancer, mRNA, miRNA, chemotherapy, survival

## Abstract

**Aim:** Cisplatin doublets are standard 1st line treatment for advanced non-small cell lung cancer (NSCLC), without accurate predictor for response and survival, but important toxicity. Our aims were to identify predictive (for response) and prognostic (for survival) biological signatures in patients with NSCLC using messenger RNAs (mRNA) and miRNA expression.

**Methods:** Patients with pathologically proven untreated NSCLC, receiving 1st line cisplatin–vinorelbine and with an assessable lesion were eligible. A bronchial biopsy was lysed into Tripure Isolation Reagent on ice, snap frozen, and stored at −80°C. mRNA expression was analyzed using microarrays Agilent Technologies. miRNA expression was assessed using TaqMan Low Density Arrays (756 human miR panel, Applied Biosystems). Validation was performed by RT-PCR on the selected genes. Survival was measured from the registration date and response assessed by WHO criteria.

**Results:** Biopsies for transcriptomic analyses were obtained from 60 consecutive patients. No statistically significant differences were observed according to the main clinical characteristics, response rate (43 vs. 41%) or survival (median 25 vs. 29 months) between derivation and validation sets. In the derivation set (*n* = 38 patients), two mRNA and one miRNA predictive signatures for response were obtained. One mRNA and one miRNA prognostic signatures were derived from the first set, allowing an adequate distinction of patients with good and poor overall and progression-free survivals. None of these signatures could be validated in the validation set (*n* = 22 patients).

**Conclusion:** In this prospective study with advanced NSCLC treated with cisplatin–vinorelbine, we were able to derive with high throughput techniques predictive and prognostic signatures based on transcriptomic analyses. However, these results could not be reproduced in an independent validation set. The role of miRNA and mRNA as predictive or prognostic factors remains a research topic and the use of high throughput technology in that context questionable. The ClinicalTrials.gov study identifier is NCT00864266 (www.clinicaltrials.gov).

## Introduction

Presently, non-small cell lung cancer (NSCLC) is the first cause of cancer death in industrialized countries. For most of the patients, the prognosis is poor (1). A few prognostic factors have been consistently reported in the literature, including performance status, gender, stage, or histology (2) but no prognostic score is allowing valid individual prognostic prediction. With a better understanding of the tumor biology and the development of high throughput techniques allowing multiple investigations on the same time, multiple prognostic biological signatures, based on messenger RNAs (mRNA) or miRNA analyses, have been proposed. However, most were constructed from retrospective studies, restricted to limited stages and surgical cases (3). Nevertheless, more than two-thirds of the patients are diagnosed at an advanced or metastatic stage for which only palliative chemotherapy can be provided. While the activity of first-line chemotherapy was demonstrated in randomized trials and meta-analyses (4), only 20–40% of the patients will exhibit an objective response. Customized therapy based on targeted agents has shown its effectiveness in tumors presenting with EGFR activating mutations (5) or ALK translocation (6). However, for most patients, there are no reliable predictive factor assessing chemosensitivity. This explains why a large number of patients are exposed uselessly to expensive and/or toxic drugs. While retrospective studies suggested that biomarkers could be helpful in predicting sensitivity to platinum derivatives (ERCC1 and XRCC1), taxanes (BRCA1), vincalcaloids (β tubulines), or gemcitabine (RRM1), two randomized trials failed to demonstrate that a genotypic approach based on ERCC1 or combined ERCC1 and RRM1 can help in predicting chemosensitivity and survival (7, 8).

The European Lung Cancer Working Party (ELCWP) initiated a prospective study aiming to find biological signatures that would predict response to chemotherapy and prognostic significance for progression-free and overall survival in advanced and metastatic NSCLC. First data of this study were previously published, concerning the miRNA analyses performed on a homogeneous derivation group of 38 patients treated with a same combination of cisplatin and vinorelbine. We were able to identify a two-miRNA signature predicting response to this chemotherapy regimen and a four-miRNA signature with prognostic value for survival (9).

The current study presents the results of our prospective study based on both mRNA and miRNA analyses and including the data from the initial derivation cohort and a validation cohort.

## Materials and Methods

To be eligible for the study, patients had to meet the following inclusion criteria: histologically confirmed NSCLC (generally, this information was available after registration due to the design of the protocol), to have a bronchial biopsy for biomarkers analysis, to be a candidate for standard first-line chemotherapy. Other key eligibility criteria were: age ≥ 18 years; presence of a measurable lesion; no prior chemotherapy; no history of cancer other than non-melanoma skin cancer, *in situ* cervical cancer or cured malignancy (interval > 5 years without recurrence). Signed informed consent had to be obtained prior to registration. The ethics committees of the participating institutions had approved the study protocol, in accordance with current legislation. The ClinicalTrials.gov study identifier is NCT00864266.

After registration at the ELCWP data center, biopsies and complete tumoral work-up, a therapeutic choice was made by the physician in charge of the patient. If the indication of chemotherapy for NSCLC was confirmed, the choice of treatment was left to the investigator, based on clinical practice guidelines of the ELCWP (10), with a preferential option for the combination of cisplatin (60 mg/m^2^ day 1) plus vinorelbine (25 mg/m^2^ days 1 and 8), every 3 weeks in order to obtain a homogeneous group for biomarkers analysis. For the present analysis, the selected group of patients required histologically confirmed NSCLC whose response to chemotherapy with cisplatin–vinorelbine was evaluable according to WHO criteria (11) and adequate biopsy obtained for transcriptomic analysis. Evaluation of response was performed every three cycles and in case of objective response, patients were treated until best response. All charts were reviewed during regular meetings by at least three independent ELCWP investigators. Patients with early progression or death due to malignant disease prior to evaluation or toxicity and treatment cessation due to toxicity were considered as treatment failures. Survival was measured from the registration date until death from any cause or last date known to be alive. Progression-free survival was measured from date of registration until date of first progression or death.

### Biopsy procedure

The procedure for collecting and processing bronchial biopsies was standardized. Any patient with pulmonary lesion consistent with the diagnosis of lung cancer and for which bronchoscopy was considered, was offered the protocol before any treatment has been applied. The sequence of diagnostic bronchoscopy was identical to a standard one, with the exception of additional samples for the study. A minimum of two tumoral biopsies were collected if the tumor was accessible during endoscopy. For each tumor biopsy, a control sample was taken in a macroscopically healthy bronchial area, remote from the tumor. Among the biopsies, the first sample was fixed in formalin and embedded in paraffin for histological diagnosis. The second one was treated for transcriptomic analyses (mRNA and miRNA) by high throughput techniques. It was directly lysed in Tripure (Roche Diagnostics, Indianapolis, IN, USA) on ice and snap frozen with liquid nitrogen. If possible, a third series of biopsies was collected and directly frozen in liquid nitrogen in order to store it in a tissue bank for further molecular biology analyses. Both series of biopsies collected for molecular biology were stored at −80°C.

### Nucleic acid isolation

This procedure allowed isolation of total RNA for both mRNAs and miRNAs expression analyses. RNA isolation was performed using the Tripure reagent (Roche Diagnostics). We added 20 μg of glycogen (Roche Diagnostics) as carrier and the separation between the organic and the aqueous phases was achieved on Phase Lock Gel (Eppendorf, Hamburg, Germany), optimizing the recovery of nucleic acids. RNA was assessed for quantity and purity on the NanoDrop ND-1000 spectrophotometer (NanoDrop Technologies, Rockland, DE, USA) and for integrity on the Agilent 2100 bioanalyser with RNA 6000 NanoAssay (Agilent Technologies, Palo Alto, CA, USA). The extracted RNAs were stored at −80°C. The RNA was used to assess expression of the mRNAs using microarrays (Agilent Technologies) and of the miRNAs by Taqman Low Density arrays (Applied Biosystem).

### Messenger RNAs expression analysis (microarrays)

Messenger RNAs were reverse-transcribed by using a T7 primer coupled with oligo-dT primers and Moloney murine leukemia virus-reverse transcriptase (MMLV-RT). The cDNAs were then *in vitro* transcribed into labeled cRNA by the T7 RNA polymerase with fluorescent nucleotides, Cy5 for samples or Cy3 for reference RNA dyes, by using the Low input RNA Fluorescent Linear Amplification Plus kit (Agilent Technologies). RNAs spike-in (Agilent Technologies) served as positive controls to monitor the whole microarray workflow (sample amplification, labeling and microarray processing). An amount of 100 ng of starting total RNA was engaged for each sample and 100 ng of pooled reference RNAs was amplified in parallel in the same experiment with the same Master-Mix. Labeled cRNA was checked for quantity and dye incorporation by the NanoDrop ND-1000 spectrophotometer (NanoDrop Technologies) and the Gaussian distribution of sample sizes was assessed by migration profiles on the Agilent 2100 bioanalyser with RNA 6000 NanoAssay (Agilent Technologies). Thereafter, the labeled cRNAs was hybridized on an Agilent oligonucleotide microarray (Two Colors Whole Human Genome 4 × 44 K arrays, Agilent Technologies) for 17 h at 65°C in a rotating oven according to the provider’s protocol (Agilent Technologies). After disassembling the hybridization chambers, the slides were washed and the signal was read by confocal laser scanning (Agilent Technologies). Grid positioning, spots localization, outliers flagging, fluorescence intensities quantification, background level assessment, and correction of the values according to the background followed by linear and Lowess data normalization were performed by using Feature Extraction software (Agilent Technologies). Quality control metrics were calculated by using this software and the arrays, which reached the 12/12 points among the 12 qualitative experimental aspects assessed (reproducibility of spike-in RNAs, maximum acceptable background, quantification of the outliers, reproducibility of the replicated spots on the arrays, etc.) were included in the analysis. Statistical analyses of microarray data were performed with the Genespring GX software (Agilent Technologies).

### miRNA expression analysis

The miRNA were reverse-transcribed and amplified by PCR using the multiplex RT TaqMan MicroRNA Low Density Array (LDA) kit (Applied Biosystem, Foster City, CA, USA). An amount of 700 ng total RNA was used for each sample. The global miRNA profiling for human miRNA (756 miR probes) was then performed by using the TaqMan LDA Human microRNA Panel (Applied Biosystem). All the quality control tests were validated: blanks and reproducibility [standard deviation of cycle threshold (CT) < 1] with the two small nucleolar house-keeping RNAs RNU48 (SNORD48) and RNU44 (SNORD44). The amount of RNA from each sample was calibrated to RNU48 that had the smallest standard deviation of all miRNA. This value gave a delta CT (ΔCT) value for each miR (miR CT value – RNU48 CT value). The average ΔCT was calculated for responders and non-responders and the delta ΔCTs (ΔΔCTs) corresponded to the difference in ΔCT between the two categories. Fold changes (FC) were calculated as 2^−ΔΔCT^ for up-regulated, as a decrease in one CT value was equivalent to a twofold increase in the starting amount of cDNA, and by 2^ΔΔCT^ for down-regulated miRNA.

### Internal validation of the genes found in microarrays analyses by RT-qPCR

The reverse transcriptase quantitative PCR (RT-qPCR) was done by TaqMan. The qPCR reaction consisted to use the Mesa Green qPCR Master-Mix Plus diluted 2×, 300 nM of forward and reverse primers, and diluted template cDNA in a range of 5 and 10×, in function of the RNA quantity assessed by the NanoDrop ND-1000 spectrophotometer. Cycling was performed at the following conditions: 15 min at 42°C, 3 min at 95°C followed by 40 rounds of 15 s at 95°C, 20 s at 60°C, and 40 s at 72°C. The assay included two no-template controls that consisted of the same samples without the reverse transcription and a control of potential non-specific amplifications by using melting curves.

### Statistical considerations

The primary objective of the study was to identify a molecular signature to predict response to chemotherapy in patients with NSCLC. The secondary objectives were to identify prognostic signatures for survival and progression-free survival. Statistical considerations are detailed at www.elcwp.org. The lack of data and the absence of guidelines in this particular setting led us to make assumptions on the study power with different scenarios. So, assuming the existence of a predictive signature, we calculated the power to detect the effect of the signature on response, with varying rates of patients with a predictive signature of response (between 10 and 50%) for standard chemotherapy and for three different values for objective response rate overall. We also targeted a response rate among patients predicted to be responders of 75% at least. The power, including a minimum of 15 patients evaluable for objective response to perform the genetic analysis in the derivation group, was more than 80% in almost all circumstances with predicted response rates above 20% (power 0.90–1.00) except in the situation of low rates (<20%, power 0.47–0.80) of patients predicted to be responders, but in this case the signature can be considered as not sensitive enough and then without clinical usefulness. According to the protocol, the signature should be confirmed in an independent validation group.

Differences between responders and non-responders according to clinical characteristics were assessed by *t*-tests or a Fisher’s Exact tests. We applied *t*-tests for comparing mRNA expression and Wilcoxon test for miRNA expression between responders and non-responders, after adjusting for multiplicity testing using the Benjamini–Hochberg (BH) method (12). False discovery rate was set to 5%. The signature for response was derived using logistic regression with stepwise variable selection. The associations between overall survival and mRNA or miRNA expression levels were estimated by using the Kaplan–Meier method and a log-rank test. Cox proportional hazard regression models were applied to estimate the hazard ratios. The signatures for overall survival were derived using Cox proportional hazards models with stepwise variable selection.

## Results

**Figure 1:**
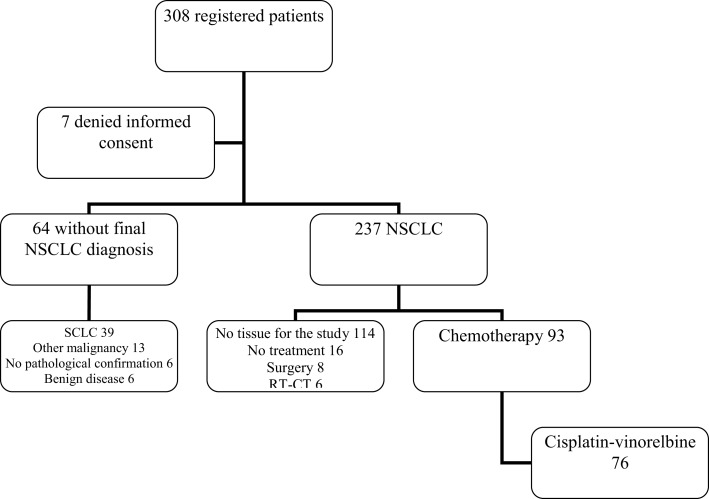
**Flow chart of registered patients**. NSCLC, non-small cell lung cancer; SCLC, small cell lung cancer; RT-CT, radiochemotherapy.

From 1/04/2009 to 12/06/2013, 308 patients, with a high suspicion of lung cancer based on lung lesion on chest CT scan, were prospectively registered (Figure 1). Seven patients further denied their initial consent, leaving 301 patients assessable for the study. In 25 cases, the diagnosis of lung cancer could not be confirmed. The two main reasons were malignant tumor from another site (*n* = 13) or a benign disease (*n* = 6). Despite the clinical suspicion, no pathologic confirmation could be obtained in six cases. A pathological diagnosis of lung cancer was found in 276 patients, either on samples obtained at bronchoscopy or during a subsequent procedure. Among them, there were 39 small cell lung cancers leaving 237 NSCLC for further analyses.

Among the 237 NSCLC, no tissue sample eligible for the genomic analyses can be obtained in 114 cases. From the remaining 123 patients, 16 did not receive any treatment (exclusive palliative care and/or rapid alteration of the general status not allowing any anticancer therapy) and 14 were treated with curative intent including a local treatment not allowing evaluation of chemotherapy activity (eight surgery and six radiochemotherapy).

**Figure 2:**
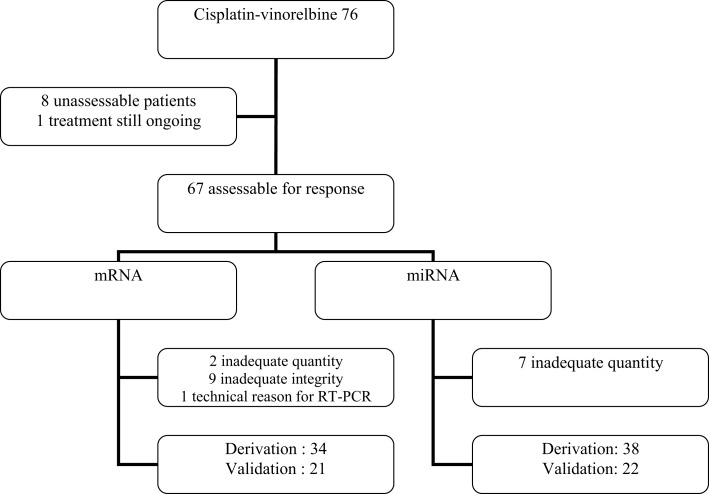
**Flow chart of the patients treated with cisplatin–vinorelbine for transcriptomic analysis**.

Among 93 patients benefiting from chemotherapy, 76 did receive a combination of cisplatin (60 mg/m^2^ day 1) and vinorelbine (25 mg/m^2^ days 1 and 8). From these 76 patients (Figure 2), eight were not assessable for response and at the time of analysis, one patient was still under therapy and not yet assessed. Sixty-seven patients were evaluable for response but only 55 samples were adequate for performing microarrays (mRNA), the other 11 showing either insufficient quantity (*n* = 2), poor quality (*n* = 9), or unassessable for technological consideration (*n* = 1). The first 34 patients were included in the derivation group and the further 21 ones in the validation one. Sixty patients could be included in the miRNA analyses, the first 38 for the derivation group and the further consecutive 22 for the validation one. In seven cases, RNA was in insufficient quantity. The main characteristics of the patients are reported in the Table 1. No statistically significant differences were observed between the derivation and validation sets concerning clinical characteristics, response rate, or survival times.

**Table 1:** **Main characteristics of the patients included in the mRNA analyses**.

	Derivation group (*N* = 38)		Validation group (*N* = 22)		*P*-value
**Gender**
Male	27	(71%)	17	(77%)	0.60
female	11	(29%)	5	(23%)	
**Age**
Mean ± STD	59 ± 11		62 ± 10		0.27
Median (min–max)	60 (33–78)		62 (45–76)		
**Karnofsky PS**
50	–	–	1	(5%)	
60	2	(5%)	1	(5%)	
70	9	(24%)	1	(5%)	
80	6	(16%)	6	(30%)	
90	17	(45%)	9	(45%)	
100	4	(11%)	2	(10%)	
Missing data			2		
Median (min–max)	90 (60–100)		90 (50–100)		0.83
**Metastases**
No	8	(21%)	7	(32%)	0.36
Yes	30	(79%)	15	(68%)	
**Histology**
Adenocarcinoma	20	(53%)	8	(36%)	0.44
Squamous	15	(39%)	12	(55%)	
NSCLC NOS	3	(8%)	2	(9%)	
**Response to chemotherapy**
No response	21	(57%)	13	(59%)	0.86
Response	16	(43%)	9	(41%)	
Unassessable	1				
**Median OS (months) (95% CI)**	25 (19–40)		29 (15–40)		0.63

*OS, overall survival; PS, performance status; NOS, not otherwise specified*.

### mRNA analyses

#### Derivation group

##### Constructing a signature predicting response to cisplatin–vinorelbine

Thirty-four patients were assessable for response and mRNA analyses in the derivation group. Among those 34 patients, we observed 14 objective responses (41%) confirmed by an independent panel of ELCWP investigators. Data were imported in Genespring as Agilent two-color data, preprocessed by Feature Extraction software with normalization and baseline to median transformation of all samples. All quality indicators appeared correct with and without baseline transformation.

We removed from the 41,093 probes the 14,523 that were not expressed in any of the samples, leaving 26,570 probes for the analyses. If we restricted to only the probes with detected intensities in all of the samples of at least one experimental condition (response/non-response), then out of the 41,093 probes, 26,567 would have been retained. If we had restricted only to the probes with detected intensities in all of the samples for both response and non-response, then out of the 41,093 probes, 24,609 probes would have been retained, but potentially interesting biological changes between response and non-response might be missed. Therefore, we have decreased the stringency of the filter such that even if the gene is only expressed (associated with detected or marginal intensity) in all of the samples in any experimental condition (response/non-response), the probe will pass the filter. So, only undetected probes were removed.

We applied *t*-tests (asymptotic *p*-value) for comparing mRNAs expression according to response, after adjusting for multiplicity testing [false discovery rate (FDR) according to BH method <0.05]. We found 115 mRNAs differentially expressed between responders and non-responders (Table 2), 50 mRNAs having a FC >2 and 19 with a FC >3 (Table 3).

**Table 2:** **List of 115 genes differentially expressed between responders and non-responders in the derivation group**.

Fold change	Probe name	Response (mean value)	No response (mean value)	Difference in mean values	Fold change (response vs. no response)	Absolute fold change	Regulation (response vs. no response)	FDR corrected *p*-value	Uncorrected *p*-value	Gene symbol
FC > 4	A_23_P38537	2.4904087	−1.35274	3.8431487	14.351689	14.351689	Up	2.16E-02	8.13E-06	KRT16
FC > 4	A_23_P23296	2.3682325	−0.70072174	3.0689542	8.391648	8.391648	Up	3.20E-02	2.91E-05	PKP1
FC > 4	A_32_P168973	2.0731359	−0.94601595	3.0191517	8.106908	8.106908	Up	3.26E-02	7.61E-05	MGC102966
FC > 4	A_24_P228149	2.0986717	−0.45802718	2.5566988	5.8835983	5.8835983	Up	3.26E-02	6.87E-05	KRT13
FC > 4	A_23_P63736	1.6036273	−0.8539755	2.4576027	5.493032	5.493032	Up	1.39E-02	3.31E-06	LOC84856
FC > 4	A_23_P93737	1.7272507	−0.36085242	2.088103	4.2518864	4.2518864	Up	4.04E-02	1.17E-04	DYNC1I1
FC > 4	A_32_P29118	1.1011099	−0.9703983	2.0715082	4.2032585	4.2032585	Up	4.92E-02	2.11E-04	SEMA3D
FC > 4	A_23_P405287	1.3622949	−0.7057522	2.068047	4.1931868	4.1931868	Up	3.20E-02	4.81E-05	SEMA3D
FC > 3	A_23_P145485	1.3383486	−0.6611513	1.9994999	3.9986138	3.9986138	Up	3.20E-02	4.41E-05	ULBP2
FC > 3	A_24_P149314	1.3553609	−0.5968727	1.9522336	3.8697317	3.8697317	Up	3.85E-02	1.10E-04	ULBP2
FC > 3	A_23_P168259	1.3069798	−0.6392596	1.9462394	3.8536868	3.8536868	Up	3.26E-02	7.40E-05	ULBP2
FC > 3	A_24_P55295	1.029862	−0.8678826	1.8977447	3.7263021	3.7263021	Up	3.26E-02	6.36E-05	GJA1
FC > 3	A_24_P260134	1.5008985	−0.35766622	1.8585647	3.626467	3.626467	Up	3.03E-02	2.48E-05	NMNAT3
FC > 3	A_23_P212665	1.3599136	−0.40204343	1.761957	3.391579	3.391579	Up	3.20E-02	3.70E-05	ABCC5
FC > 3	A_24_P402588	0.9555351	−0.75297946	1.7085146	3.2682414	3.2682414	Up	4.77E-02	1.90E-04	BCL11A
FC > 3	A_24_P15621	1.1812744	−0.41237277	1.5936472	3.0181139	3.0181139	Up	4.84E-02	2.00E-04	SLC6A10P
FC > 2	A_24_P50890	1.0291404	−0.41876024	1.4479005	2.7281077	2.7281077	Up	3.74E-02	1.03E-04	PVRL1
FC > 2	A_23_P120467	1.2124797	−0.22860909	1.4410888	2.7152572	2.7152572	Up	3.20E-02	4.77E-05	ZFP64
FC > 2	A_23_P209904	0.95239705	−0.44006458	1.3924617	2.6252625	2.6252625	Up	3.26E-02	7.05E-05	GPC1
FC > 2	A_23_P211401	0.8455327	−0.5219133	1.367446	2.580134	2.580134	Up	3.20E-02	3.70E-05	KREMEN1
FC > 2	A_23_P76034	0.97714347	−0.36702603	1.3441695	2.53884	2.53884	Up	3.26E-02	6.86E-05	PVRL1
FC > 2	A_23_P365785	0.65030813	−0.6916805	1.3419886	2.5350049	2.5350049	Up	3.52E-02	8.95E-05	GRHL1
FC > 2	A_24_P351283	1.0064123	−0.33283657	1.3392489	2.5301955	2.5301955	Up	2.54E-02	1.34E-05	MREG
FC > 2	A_32_P78943	1.0684785	−0.14730063	1.2157791	2.3226616	2.3226616	Up	4.73E-02	1.87E-04	
FC > 2	A_24_P371399	1.0383815	−0.17136136	1.2097428	2.312964	2.312964	Up	3.20E-02	3.80E-05	C3orf58
FC > 2	A_23_P210763	0.8555546	−0.28793192	1.1434865	2.2091424	2.2091424	Up	3.26E-02	7.43E-05	JAG1
FC > 2	A_23_P144134	0.92170376	−0.19656532	1.1182691	2.1708636	2.1708636	Up	4.31E-02	1.45E-04	C3orf58
FC > 2	A_24_P567454	0.9409114	−0.1519253	1.0928367	2.1329303	2.1329303	Up	3.13E-03	2.66E-07	RNF168
FC > 2	A_32_P26443	0.66768515	−0.4232059	1.0908911	2.1300557	2.1300557	Up	3.20E-02	3.53E-05	C3orf21
FC > 2	A_23_P18196	0.67252	−0.3965082	1.0690281	2.0980196	2.0980196	Up	3.26E-02	6.66E-05	RFC4
FC > 2	A_24_P46171	0.78893715	−0.26471114	1.0536482	2.0757723	2.0757723	Up	3.20E-02	4.65E-05	PIGX
FC > 2	A_23_P354170	0.74245876	−0.30971533	1.0521741	2.0736525	2.0736525	Up	1.24E-02	2.33E-06	PIGX
FC > 2	A_24_P123119	0.6953678	−0.35124567	1.0466135	2.0656753	2.0656753	Up	3.74E-02	1.02E-04	EHHADH
FC > 2	A_24_P123190	0.6343807	−0.3929577	1.0273384	2.0382605	2.0382605	Up	4.73E-02	1.85E-04	PLD1
	A_23_P108948	0.7275434	−0.25510627	0.9826497	1.9760914	1.9760914	Up	3.26E-02	6.88E-05	MREG
	A_24_P23400	0.8408562	−0.13957612	0.98043233	1.9730566	1.9730566	Up	3.26E-02	7.49E-05	SLC6A8
	A_24_P111737	0.8168281	−0.14033504	0.9571631	1.9414884	1.9414884	Up	3.26E-02	6.39E-05	ATP11B
	A_32_P195291	0.7964409	−0.15760839	0.9540493	1.9373026	1.9373026	Up	3.20E-02	3.34E-05	RNF168
	A_23_P69121	0.82585055	−0.10896964	0.9348202	1.9116523	1.9116523	Up	4.73E-02	1.83E-04	SIAH2
	A_23_P400794	0.6963362	−0.23828867	0.9346249	1.9113936	1.9113936	Up	3.26E-02	6.38E-05	FBXO45
	A_24_P406245	0.71501625	−0.21661237	0.9316286	1.907428	1.907428	Up	3.20E-02	4.06E-05	PMS2L2
	A_24_P340428	0.67214394	−0.25692314	0.9290671	1.9040444	1.9040444	Up	1.39E-02	3.66E-06	ATP11B
	A_23_P16944	0.77089965	−0.14973187	0.9206315	1.8929437	1.8929437	Up	4.60E-02	1.71E-04	SDC1
	A_23_P31135	0.5873326	−0.32817858	0.9155112	1.8862373	1.8862373	Up	4.07E-02	1.26E-04	ACAT2
	A_23_P69249	0.67190737	−0.23103127	0.9029386	1.8698708	1.8698708	Up	2.59E-02	1.54E-05	ACTL6A
	A_23_P69437	0.58162504	−0.31927863	0.9009037	1.8672353	1.8672353	Up	4.73E-02	1.85E-04	YEATS2
	A_32_P97192	0.6328155	−0.2644442	0.8972597	1.8625249	1.8625249	Up	3.26E-02	5.55E-05	PMS2L2
	A_23_P5936	0.4895308	−0.39759988	0.8871307	1.8494941	1.8494941	Up	4.31E-02	1.41E-04	C20orf117
	A_23_P345710	0.7339968	−0.15165368	0.8856505	1.8475975	1.8475975	Up	3.13E-03	1.65E-07	C3orf21
	A_23_P134295	0.5280118	−0.35337663	0.8813884	1.8421474	1.8421474	Up	3.26E-02	7.37E-05	NUDT1
	A_24_P291588	0.6312908	−0.24706182	0.87835264	1.8382751	1.8382751	Up	2.41E-02	9.97E-06	DVL3
	A_23_P358221	0.68547153	−0.1714746	0.8569461	1.8112003	1.8112003	Up	1.24E-02	2.09E-06	UBXN7
	A_23_P119084	0.54716736	−0.3036831	0.85085046	1.8035638	1.8035638	Up	4.81E-02	1.97E-04	ZNF551
	A_23_P416142	0.6109084	−0.22270343	0.83361185	1.7821414	1.7821414	Up	2.54E-02	1.29E-05	DLG1
	A_23_P212522	0.6316152	−0.19792378	0.829539	1.7771174	1.7771174	Up	2.79E-02	2.00E-05	ATP11B
	A_23_P212034	0.63048804	−0.19111416	0.8216022	1.7673677	1.7673677	Up	4.33E-02	1.54E-04	DLG1
	A_24_P944444	0.51009727	−0.29740423	0.8075015	1.7501779	1.7501779	Up	3.20E-02	3.09E-05	MAPKBP1
	A_23_P395075	0.58912766	−0.20520839	0.7943361	1.734279	1.734279	Up	4.81E-02	1.94E-04	KDM3A
	A_24_P166012	0.55840063	−0.23510353	0.7935042	1.7332793	1.7332793	Up	3.03E-02	2.51E-05	DCUN1D1
	A_23_P155316	0.48434967	−0.30781966	0.79216933	1.7316763	1.7316763	Up	4.31E-02	1.43E-04	NCBP2
	A_32_P16258	0.6029671	−0.17862633	0.78159344	1.7190285	1.7190285	Up	4.92E-02	2.13E-04	EXOC6B
	A_23_P144151	0.65791667	−0.10735836	0.765275	1.6996939	1.6996939	Up	3.52E-02	9.10E-05	
	A_23_P18102	0.40448546	−0.3493367	0.7538222	1.6862544	1.6862544	Up	3.20E-02	5.00E-05	SENP5
	A_23_P218884	0.48295045	−0.27010906	0.7530595	1.6853632	1.6853632	Up	3.20E-02	4.32E-05	DVL3
	A_32_P222515	0.5415697	−0.18197708	0.7235468	1.6512365	1.6512365	Up	3.26E-02	6.53E-05	UBXN7
	A_23_P258037	0.5379724	−0.17757766	0.71555007	1.6421092	1.6421092	Up	3.20E-02	5.06E-05	KDM3A
	A_24_P105761	0.52657336	−0.18410102	0.7106744	1.636569	1.636569	Up	4.73E-02	1.80E-04	KDM3A
	A_23_P110062	0.51333207	−0.19231682	0.7056489	1.6308781	1.6308781	Up	4.07E-02	1.29E-04	EIF2B5
	A_24_P210675	0.45133168	−0.24289604	0.6942277	1.618018	1.618018	Up	4.92E-02	2.13E-04	NDE1
	A_23_P29504	0.55088145	−0.11576251	0.666644	1.5873761	1.5873761	Up	3.26E-02	7.21E-05	SENP5
	A_24_P76666	0.35812706	−0.29609442	0.6542215	1.5737665	1.5737665	Up	3.20E-02	4.38E-05	CSNK2A1
	A_23_P431587	0.36161706	−0.26871416	0.6303312	1.5479203	1.5479203	Up	4.07E-02	1.24E-04	VPS8
	A_32_P32653	0.41394213	−0.21419649	0.6281386	1.5455695	1.5455695	Up	3.60E-02	9.62E-05	SENP5
	A_23_P101342	0.488292	−0.1337074	0.6219994	1.5390066	1.5390066	Up	2.72E-02	1.84E-05	ATG4D
	A_23_P44956	0.43706688	−0.16102271	0.5980896	1.5137107	1.5137107	Up	3.80E-02	1.07E-04	RPL35A
	A_32_P32179	0.4339779	−0.15347187	0.5874498	1.5025883	1.5025883	Up	2.54E-02	1.29E-05	
	A_23_P427622	0.5676906	0.013194157	0.55449647	1.468656	1.468656	Up	4.81E-02	1.96E-04	UNQ1887
	A_23_P110076	0.33755684	−0.20134711	0.53890395	1.4528683	1.4528683	Up	4.34E-02	1.57E-04	WDR53
	A_32_P132477	0.38293335	−0.15272838	0.5356617	1.4496069	1.4496069	Up	4.07E-02	1.20E-04	
	A_23_P346206	0.36038876	−0.16280492	0.52319366	1.4371331	1.4371331	Up	3.52E-02	8.74E-05	RAE1
	A_23_P317854	0.29310012	−0.1636454	0.4567455	1.3724424	1.3724424	Up	3.74E-02	1.04E-04	MED26
	A_24_P225907	−0.3019109	0.15964396	−0.46155488	−1.3770251	1.3770251	Down	4.07E-02	1.21E-04	DPH3
	A_24_P205230	−0.35831454	0.16462934	−0.52294385	−1.4368843	1.4368843	Down	4.07E-02	1.26E-04	RNASEK
	A_24_P166094	−0.4055858	0.13177143	−0.5373572	−1.4513115	1.4513115	Down	4.44E-02	1.62E-04	ARFIP1
	A_23_P205913	−0.42380837	0.11410071	−0.5379091	−1.4518667	1.4518667	Down	4.58E-02	1.69E-04	SLC24A1
	A_23_P152066	−0.46616426	0.09878205	−0.5649463	−1.4793324	1.4793324	Down	3.13E-03	3.53E-07	UBR1
	A_23_P58002	−0.41184562	0.1589065	−0.57075214	−1.4852977	1.4852977	Down	4.31E-02	1.46E-04	TCTA
	A_24_P71153	−0.37698016	0.19527814	−0.5722583	−1.4868492	1.4868492	Down	3.45E-02	8.32E-05	PAFAH2
	A_23_P77562	−0.37067208	0.2450805	−0.6157526	−1.5323571	1.5323571	Down	3.26E-02	5.87E-05	TMEM219
	A_23_P412392	−0.29659137	0.33060193	−0.62719333	−1.5445572	1.5445572	Down	3.20E-02	4.44E-05	SEC22B
	A_24_P551302	−0.38215917	0.2763248	−0.658484	−1.5784231	1.5784231	Down	3.26E-02	6.83E-05	
	A_23_P40866	−0.4178533	0.25824612	−0.6760994	−1.597814	1.597814	Down	3.35E-02	7.95E-05	ZBTB20
	A_23_P72503	−0.4167065	0.27814037	−0.69484687	−1.6187127	1.6187127	Down	3.03E-02	2.37E-05	KLHL2
	A_24_P823708	−0.44348216	0.27844223	−0.7219244	−1.6493807	1.6493807	Down	3.20E-02	5.03E-05	LOC728855
	A_23_P503182	−0.49898216	0.23469424	−0.73367643	−1.6628712	1.6628712	Down	3.20E-02	4.01E-05	ABR
	A_32_P28476	−0.5130292	0.2420673	−0.75509655	−1.6877445	1.6877445	Down	3.09E-02	2.68E-05	
	A_32_P193091	−0.55347806	0.28480965	−0.8382877	−1.7879268	1.7879268	Down	4.33E-02	1.51E-04	
	A_23_P12463	−0.6446937	0.28819558	−0.9328892	−1.9090954	1.9090954	Down	4.31E-02	1.40E-04	QSOX1
	A_23_P108751	−0.538324	0.45318156	−0.99150556	−1.9882588	1.9882588	Down	3.58E-02	9.43E-05	FHL2
FC > 2	A_23_P150325	−0.6769149	0.36179325	−1.0387081	−2.054387	2.054387	Down	4.07E-02	1.27E-04	TMEM133
FC > 2	A_23_P157879	−0.8153628	0.28951484	−1.1048777	−2.1508064	2.1508064	Down	4.33E-02	1.52E-04	FCN1
FC > 2	A_24_P152649	−0.6403896	0.48672542	−1.127115	−2.1842153	2.1842153	Down	4.31E-02	1.41E-04	LOC644189
FC > 2	A_23_P149562	−0.627059	0.5123564	−1.1394154	−2.2029173	2.2029173	Down	4.85E-02	2.03E-04	ARHGAP29
FC > 2	A_24_P269006	−0.7269329	0.4633929	−1.1903257	−2.2820425	2.2820425	Down	4.33E-02	1.55E-04	ALDH7A1
FC > 2	A_32_P486620	−0.9032537	0.303291	−1.2065446	−2.3078423	2.3078423	Down	4.66E-02	1.75E-04	IGSF22
FC > 2	A_23_P94819	−0.8477721	0.36849847	−1.2162706	−2.3234532	2.3234532	Down	1.97E-02	5.94E-06	RPH3AL
FC > 2	A_23_P201386	−0.72164166	0.49630338	−1.2179451	−2.3261516	2.3261516	Down	2.59E-02	1.65E-05	DDAH1
FC > 2	A_24_P333571	−0.9850959	0.32136387	−1.3064598	−2.4733386	2.4733386	Down	3.52E-02	8.98E-05	
FC > 2	A_23_P116614	−1.1228174	0.27101618	−1.3938336	−2.6277602	2.6277602	Down	4.33E-02	1.54E-04	ME3
FC > 2	A_23_P105227	−1.2048123	0.2538056	−1.4586179	−2.7484493	2.7484493	Down	3.26E-02	6.51E-05	ME3
FC > 2	A_23_P148753	−1.0244241	0.4529644	−1.4773885	−2.7844424	2.7844424	Down	4.92E-02	2.12E-04	PLEKHA6
FC > 2	A_23_P15272	−1.0517842	0.45105672	−1.5028409	−2.8340023	2.8340023	Down	3.52E-02	9.15E-05	ABCC6
FC > 3	A_23_P431268	−1.0362521	0.62329584	−1.659548	−3.1591754	3.1591754	Down	2.16E-02	7.96E-06	PLEKHA6
FC > 3	A_23_P10182	−1.0682763	0.7183502	−1.7866265	−3.450072	3.450072	Down	2.59E-02	1.60E-05	ACOX2
FC > 4	A_23_P394246	−1.4391525	0.9436475	−2.3828	−5.2154803	5.2154803	Down	3.26E-02	6.28E-05	GPR81

**Table 3:** **Number of genes differentially expressed in responders and non-responders in the derivation group**.

	Without FDR correction	With FDR < 0.05
All fold change	26570	115
Fold change > 1.1	15789	115
Fold change > 1.5	2423	103
Fold change > 2.0	516	50
Fold change > 3.0	115	19

*FDR, false discovery rate (according to the Benjamini–Hochberg method)*.

We restricted further analyses to the mRNAs with a fold change of at least two. Among the 50 mRNAs differentially expressed, 34 were up-regulated and 16 down-regulated in non-responders in comparison with responders. We calculated the area under the ROC curve for each mRNA to predict response (genes detailed in Table 4). After a stepwise variable selection on the 50 mRNAs, two variables (FCN1 and RNF168) were retained. The area under the ROC curve, when including both FCN1 and RNF168, was 0.97 [95% confidence interval (CI) 0.91–1]. It allowed constructing a signature predicting response to cisplatin–vinorelbine summarized as −2*FCN1 + 5*RNF168. At the best threshold of >2.3, the signature had 93% (13/14) sensitivity, 100% (20/20) specificity, 100% (13/13) positive predictive value, and 95% (20/21) negative predictive value (Table 5).

**Table 4:** **Area under ROC curve for the 50 genes differentially expressed between responders and non-responders with a fold change > 2**.

ProbeName	GeneSymbol	Area under ROC curve	Description
A_23_P157879	FCN1	0.86	Homo sapiens ficolin (collagen/fibrinogen domain containing) 1 (FCN1), mRNA [NM_002003]
A_23_P144134	C3orf58	0.78	Homo sapiens chromosome 3 open reading frame 58 (C3orf58), transcript variant 1, mRNA [NM_173552]
A_23_P394246	GPR81	0.87	Probable G-protein coupled receptor 81 (G-protein coupled receptor 104) [Source:UniProtKB/Swiss-Prot;Acc:Q9BXC0] [ENST00000432564]
A_23_P431268	PLEKHA6	0.90	Homo sapiens pleckstrin homology domain containing, family A member 6 (PLEKHA6), mRNA [NM_014935]
A_24_P269006	ALDH7A1	0.86	Homo sapiens aldehyde dehydrogenase 7 family, member A1 (ALDH7A1), mRNA [NM_001182]
A_23_P145485	ULBP2	0.89	Homo sapiens UL16 binding protein 2 (ULBP2), mRNA [NM_025217]
A_24_P228149	KRT13	0.85	Homo sapiens keratin 13 (KRT13), transcript variant 2, mRNA [NM_002274]
A_23_P23296	PKP1	0.88	Homo sapiens plakophilin 1 (ectodermal dysplasia/skin fragility syndrome) (PKP1), transcript variant 1b, mRNA [NM_000299]
A_32_P78943		0.78	CD025760 Human CD34(ESTs from primary hematopoietic stem-progenitor cells Homo sapiens cDNA 3′, mRNA sequence [GD161310]
A_23_P105227	ME3	0.85	Homo sapiens malic enzyme 3, NADP(+)-dependent, mitochondrial (ME3), nuclear gene encoding mitochondrial protein, transcript variant 2, mRNA [NM_001014811]
A_23_P10182	ACOX2	0.90	Homo sapiens acyl-Coenzyme A oxidase 2, branched chain (ACOX2), mRNA [NM_003500]
A_24_P402588	BCL11A	0.86	Homo sapiens B-cell CLL/lymphoma 11A (zinc finger protein) (BCL11A), transcript variant 2, mRNA [NM_018014]
A_23_P94819	RPH3AL	0.91	Homo sapiens rabphilin 3A-like (without C2 domains) (RPH3AL), mRNA [NM_006987]
A_23_P211401	KREMEN1	0.85	Homo sapiens kringle containing transmembrane protein 1 (KREMEN1), transcript variant 3, mRNA [NM_001039570]
A_24_P333571		0.87	Rho GTPase-activating protein 29 (Rho-type GTPase-activating protein 29)(PTPL1-associated RhoGAP protein 1) [Source:UniProtKB/Swiss-Prot;Acc:Q52LW3] [ENST00000370217]
A_23_P63736	LOC84856	0.91	Homo sapiens hypothetical LOC84856 (LOC84856), non-coding RNA [NR_026827]
A_23_P150325	TMEM133	0.87	Homo sapiens transmembrane protein 133 (TMEM133), mRNA [NM_032021]
A_23_P209904	GPC1	0.85	Homo sapiens glypican 1 (GPC1), mRNA [NM_002081]
A_24_P567454	RNF168	0.94	E3 ubiquitin-protein ligase RNF168 (EC 6.3.2.-)(RING finger protein 168) [Source:UniProtKB/Swiss-Prot;Acc:Q8IYW5] [ENST00000318037]
A_23_P38537	KRT16	0.90	Homo sapiens keratin 16 (KRT16), mRNA [NM_005557]
A_23_P365785	GRHL1	0.86	Homo sapiens grainyhead-like 1 (Drosophila) (GRHL1), mRNA [NM_198182]
A_24_P260134	NMNAT3	0.85	Homo sapiens nicotinamide nucleotide adenylyltransferase 3 (NMNAT3), mRNA [NM_178177]
A_23_P15272	ABCC6	0.87	Homo sapiens ATP-binding cassette, sub-family C (CFTR/MRP), member 6 (ABCC6), transcript variant 2, mRNA [NM_001079528]
A_24_P123190	PLD1	0.87	Homo sapiens phospholipase D1, phosphatidylcholine-specific (PLD1), transcript variant 1, mRNA [NM_002662]
A_32_P168973	MGC102966	0.86	Homo sapiens keratin 16 pseudogene (MGC102966), non-coding RNA [NR_029393]
A_23_P201386	DDAH1	0.90	Homo sapiens dimethylarginine dimethylaminohydrolase 1 (DDAH1), transcript variant 1, mRNA [NM_012137]
A_32_P486620	IGSF22	0.84	Homo sapiens immunoglobulin superfamily, member 22 (IGSF22), mRNA [NM_173588]
A_24_P55295	GJA1	0.89	Homo sapiens gap junction protein, alpha 1, 43kDa (GJA1), mRNA [NM_000165]
A_32_P29118	SEMA3D	0.85	Homo sapiens sema domain, immunoglobulin domain (Ig), short basic domain, secreted, (semaphorin) 3D (SEMA3D), mRNA [NM_152754]
A_24_P152649	LOC644189	0.87	PREDICTED: Homo sapiens similar to peroxisomal long-chain acyl-coA thioesterase (LOC644189), miscRNA [XR_016949]
A_23_P168259	ULBP2	0.87	Homo sapiens UL16 binding protein 2 (ULBP2), mRNA [NM_025217]
A_24_P149314	ULBP2	0.86	Homo sapiens UL16 binding protein 2 (ULBP2), mRNA [NM_025217]
A_23_P120467	ZFP64	0.87	Homo sapiens zinc finger protein 64 homolog (mouse) (ZFP64), transcript variant 4, mRNA [NM_199427]
A_24_P123119	EHHADH	0.89	Homo sapiens enoyl-Coenzyme A, hydratase/3-hydroxyacyl Coenzyme A dehydrogenase (EHHADH), transcript variant 1, mRNA [NM_001966]
A_24_P351283	MREG	0.90	Homo sapiens melanoregulin (MREG), mRNA [NM_018000]
A_24_P15621	SLC6A10P	0.82	Homo sapiens solute carrier family 6 (neurotransmitter transporter, creatine), member 10 (pseudogene) (SLC6A10P) on chromosome 16 [NR_003083]
A_24_P50890	PVRL1	0.83	Homo sapiens poliovirus receptor-related 1 (herpesvirus entry mediator C) (PVRL1), transcript variant 1, mRNA [NM_002855]
A_24_P371399	C3orf58	0.81	Homo sapiens chromosome 3 open reading frame 58 (C3orf58), transcript variant 1, mRNA [NM_173552]
A_23_P148753	PLEKHA6	0.83	Homo sapiens pleckstrin homology domain containing, family A member 6 (PLEKHA6), mRNA [NM_014935]
A_32_P26443	C3orf21	0.86	Uncharacterized protein C3orf21 [Source:UniProtKB/Swiss-Prot;Acc:Q8NBI6] [ENST00000310380]
A_23_P405287	SEMA3D	0.87	Homo sapiens sema domain, immunoglobulin domain (Ig), short basic domain, secreted, (semaphorin) 3D (SEMA3D), mRNA [NM_152754]
A_23_P149562	ARHGAP29	0.84	Homo sapiens Rho GTPase-activating protein 29 (ARHGAP29), mRNA [NM_004815]
A_23_P210763	JAG1	0.85	Homo sapiens jagged 1 (Alagille syndrome) (JAG1), mRNA [NM_000214]
A_23_P116614	ME3	0.84	Homo sapiens malic enzyme 3, NADP(+)-dependent, mitochondrial (ME3), nuclear gene encoding mitochondrial protein, transcript variant 2, mRNA [NM_001014811]
A_23_P93737	DYNC1I1	0.83	Homo sapiens dynein, cytoplasmic 1, intermediate chain 1 (DYNC1I1), transcript variant 1, mRNA [NM_004411]
A_23_P18196	RFC4	0.86	Homo sapiens replication factor C (activator 1) 4, 37kDa (RFC4), transcript variant 1, mRNA [NM_002916]
A_23_P76034	PVRL1	0.88	Homo sapiens poliovirus receptor-related 1 (herpesvirus entry mediator C) (PVRL1), transcript variant 3, mRNA [NM_203286]
A_23_P354170	PIGX	0.93	Homo sapiens phosphatidylinositol glycan anchor biosynthesis, class X (PIGX), transcript variant 2, mRNA [NM_017861]
A_23_P212665	ABCC5	0.87	Homo sapiens ATP-binding cassette, sub-family C (CFTR/MRP), member 5 (ABCC5), transcript variant 1, mRNA [NM_005688]
A_24_P46171	PIGX	0.87	Homo sapiens phosphatidylinositol glycan anchor biosynthesis, class X (PIGX), transcript variant 2, mRNA [NM_017861]

**Table 5:** **Results of the stepwise regression and construction of signature predicting response in patients treated with cisplatin–vinorelbine based on mRNA analyses**.

Genes with fold change > 2 (*n* = 50)			

	Estimate	Standard error	*p*
FCN1	−2.2393	1.0798	0.0381
RNF168	5.5365	2.4143	0.0218
Signature:			
−2*FCN1 + 5*RNF168 > 2.3 → response			
−2*FCN1 + 5*RNF168 < 2.3 → no response			

**Genes with fold change > 3 (*n* = 19)**			

	**Estimate**	**Standard error**	***p***

KRT16	0.7616	0.3250	0.0191
SEMA3D	1.3641	0.6383	0.0326
Signature:			
KRT16 + 2*SEMA3D > −2 → response			
KRT16 + 2*SEMA3D < −2 → no response			

As a FC >3 is another limit accepted in the literature, we performed the analysis with the same methodology, restricted to the 19 mRNAs with a FC above 3. Three were down-regulated and 16 up-regulated in non-responders in comparison with responders. After a stepwise variable selection on the 19 mRNAs, two variables (KRT16 and SEMA3D) were retained. KRT16 had a FC of 14, SEMA3D a FC of 4 (Table 2). The area under the ROC curve, when including both KRT16 and SEMA3D, was 0.95 (95% CI 0.89–1). The signature (KRT16 + 2*SEMA3D) allowed predicting response with 100% (14/14) sensitivity, 100% negative predictive value (14/14), 70% specificity (14/20), and 70% (14/20) positive predictive value with a best cut-off of −2 (Table 5).

The mRNAs found in the two signatures were further validated in the same patients by RT-qPCR (Table 6). The following forward (Fw) and reverse (Rev) primers were used for the selected genes FCN1 (Fw: TAGAGCTGGGGGACTCTTCA; Rev: CCACCTTCACCTCTGGACAT), RNF168 (Fw: TTGGCAGAGGAGGAAGAAGA; Rev: TCAAGGGAGAAGCCGAGATA), KRT16 (Fw: GTGAAGATCCGTGACTGGTA; Rev: GCAATGATCTTGTTCCTCAG), and SEMA3D (Fw: TGGAATTGTCTCTGAAGCAGC; Rev: TGCGCAAGGTTTCCCATAAG). They were for the reference genes HPRT (Fw: GGTCAGGCAGTATAATCCAAAG; Rev: AAGGGCATATCCTACAACAAAC) and β-actin (Fw: CGCCGCCAGCTCACCATG; Rev: CACGATGGAGGGGAAGACGG). Using this methodology, we could confirm in that group of patients that the four genes were differentially expressed with a high statistical significance between responders and non-responders. The *p*-value were respectively 0.006 (FCN1), 0.008 (RNF168), 0.002 (KRT16), and 0.005 (SEMA3D). When normalized with the reference gene HPRT, all except RNF168 retained their statistical significance with respective *p*-value of <0.01 (FCN1-HPRT), 0.68 (RNF168-HPRT), 0.002 (KRT16-HPRT), and 0.06 (SEMA3D-HPRT).

**Table 6:** **Internal validation of the four genes found in the two predictive signatures for response by RT-qPCR in the derivation set**.

Variable	Non-responders			Responders			*P*-value
	N	Mean ΔCT	SD	N	Mean ΔCT	SD	
HPRT	17	27.1154287	1.0258238	14	26.1534160	0.9717660	0.027
FCN1	17	28.8819719	1.1437691	14	30.2759826	1.2235700	0.006
RNF168	17	26.9853971	0.9217302	14	25.8188423	1.1153445	0.008
KRT16	16	32.1516412	2.8771146	14	27.3871707	3.1949645	0.002
Sema3d	17	32.8827111	1.5138481	14	30.5694798	1.9132922	0.005
**HPRT AS REFERENCE GENE**							
FCN1	17	1.7665432	1.3993750	14	4.1225666	1.4610938	<0.001
RNF168	17	−0.1300316	0.6584257	14	−0.3345736	0.7560190	0.68
KRT16	16	5.0245187	2.6242224	14	1.2337547	2.6897044	0.002
Sema3D	17	5.7672824	1.9953494	14	4.4160639	1.4020225	0.06

*The tumoral expression of the selected mRNA included in the predictive signatures for response to cisplatin–vinorelbine was tested by RT-qPCR, and normalized with the HPRT reference gene*.

We measured the diagnostic performance of both signatures by comparing their area under the ROC curve. No statistical difference was noted (*p* = 0.58).

##### Constructing a prognostic signature for survival and progression-free survival

**Figure 3:**
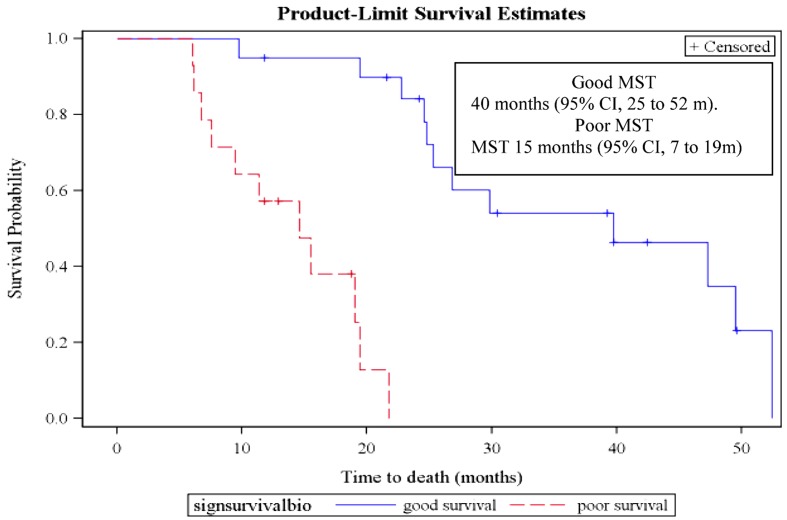
**Overall survival curves according to the mRNA prognostic signature in the derivation group**. MST, median survival time. Patients from the derivation group could be dichotomized according to the mRNA prognostic signature (−3*KRT16 + 2*ULBP2). A value >1 is predicting a poor overall survival while a value <1 is associated with a good overall survival.

Using the same statistical methodology, we constructed a signature specifically predicting overall survival using the mRNAs with a FC >3. The signature included two mRNAs (KRT16 and ULBP2) and was designed as −3*KRT16 + 2*ULBP2. At the best cut-off of 1, it allowed distinguishing patients with poor and good overall survival (HR 22.2, 95% CI 4.6–107.7; log-rank and Wilcoxon test *p* < 0.001) (Figure 3, Table 7). Respective median survival times were for the “good overall survival group” of 40 months (95% CI 25–52 months) and for the “poor overall survival group” of 15 months (95% CI 7–19 months). The same signature was statistically significantly predicting progression-free survival (HR 3.8, 95% CI 1.5–9.3; *p* < 0.001). Respective median progression-free survival times were for the “good overall survival group” of 18.6 months (95% CI, 12.3–27.3 months) and for the “poor overall survival group” of 8.1 months (95% CI, 5.8–15.5 months).

**Table 7:** **Results of the stepwise Cox’s proportional hazards model regression and construction of signature predicting survival in patients treated with cisplatin–vinorelbine, based on mRNA analyses in the derivation set**.

Genes with fold change > 3 (*n* = 19)			
	**Estimate**	**Standard error**	***p***

KRT16	−0.55394	0.16090	0.0006
ULBP2	0.39724	0.18114	0.0283
Signature:			
−3*KRT16 + 2*ULBP2 > 1 → poor overall survival			
−3*KRT16 + 2*ULBP2 < 1 → good overall survival			

#### Validation group

Reverse transcriptase quantitative PCR for the five genes included in the two predictive signatures and the one prognostic signature (FCN1, RNF168, KRT16, SEMA3D, and ULBP2) were performed on 22 samples from 22 patients treated with cisplatin–vinorelbine. One sample was excluded as the CT values for the two reference genes (HPRT and β-actin) were above 32, leaving 21 patients assessable for validating the signatures. Nine objective responses were observed. Whatever correcting for actin or HPRT, no gene was anymore differentially expressed with statistical significance between responders and non-responders (Table 8). However, we observed a statistically significant difference between the 12 non-responders and the 9 responders in the expression of the two reference genes actin (*t*-test *p* < 0.001, Wilcoxon *p* 0.046) and HPRT (*t*-test *p* 0.005, Wilcoxon *p* 0.056).

**Table 8:** **mRNA signatures for response to chemotherapy (validation set): differential expression of the genes found in the signatures between responders and non-responders**.

Variable	Non-responders (*n* = 12)		Responders (*n* = 9)		*p* (*t*-Test)	*p* (Wilcoxon)
	Mean ΔCT	SD	Mean ΔCT	SD
**β-ACTIN AS THE REFERENCE GENE**						
FCN1	1.0017325	1.5452500	−0.3936098	2.0563888	0.09	0.16
RNF168	−5.0100427	1.2120509	−5.7443545	1.2098927	0.19	0.18
KRT16	−1.6581380	2.5237281	−0.4743034	3.2727341	0.36	0.38
SEMA3D	4.5458862	2.5837169	5.2979321	2.1402825	0.49	0.55
ULBP2	1.7438450	2.1160217	0.8658941	3.2716446	0.46	0.65
**HPRT AS THE REFERENCE GENE**						
FCN1	5.6594930	2.1299628	4.3520158	2.8689951	0.24	0.35
RNF168	−0.3522822	0.8420686	−0.9987289	0.5571078	0.06	0.10
KRT16	2.9996225	2.2686257	4.2713223	2.4994064	0.24	0.25
SEMA3D	9.2036467	2.6198815	10.0435577	2.3809662	0.46	0.51
ULBP2	6.4016054	1.6569804	5.6115197	2.3782401	0.38	0.42

As no gene could be validated, we repeated the same microarray procedure for selecting variables in the 21 patients included in the validation set and tested by RT-qPCR. From the 41,093 probes, 25,693 allowed detection of a signal in at least all of the patients in one group (response/non-response). When applying *t*-tests (asymptotic *p*-value) for comparing mRNAs expression according to response, after adjusting for multiplicity testing (FDR < 0.05), no gene could be retained. We further tested both predictive and prognostic signature. None of them retained their statistical significance in the validation group, with *p*-values of 0.20 (signature for response) and 0.27 (signature for survival), respectively.

As an exploratory analysis, we also looked at genes differentially expressed between responders and non-responders, in common between the derivation and validation groups. For this purpose, we performed asymptotic *t*-tests without FDR application. Three thousand nine hundred ninety-four and 4597 genes were retained respectively in the derivation and validation sets, of which 402 were in common in both sets. Only 153 of them are regulated in the same direction, but only 10 probes had a differential expression between responders and non-responders at an FDR uncorrected *p*-value <0.01 in both sets and only one with a FC >2 in both sets. They are KCTD1, ALDH3B2, IQCB1, CHCHD6, RMND5A, NRP1 (transcript variant 3), RPS6KA5, C17orf69, NRP1 (transcript variant 1), and IQCB2P.

### miRNA analyses

#### Derivation group

##### Constructing signatures predicting response to cisplatin–vinorelbine and prognostic for survival

The results concerning the development of predictive and prognostic signatures based on tumoral miRNAs in the derivation group have been previously published (9). Briefly, miRNAs analyses were performed in 38 patients of whom one was not assessable for response. Sixteen objective responses (43%) were observed. Of the 756 miRNAs, 396 had a CT > 32 in all patients and were excluded from the analysis. Thirty-eight miRNAs were differentially expressed between responders and non-responders with a *p*-value < 0.05. If we applied the BH method for controlling the FDR for multiple testing, none of them could be retained. However, without FDR selection, a two miRNAs signature was designed: −4*hsa-miR-149 + 3*hsa-miR-375 with a score above −6 predicting response with an area under the ROC curve of 0.90 and 88% sensitivity, 81% specificity, 78% positive, and 89% negative predictive values.

To challenge the signature, we analyzed the prognostic value of miRNAs for overall survival among 38 patients, 25 being dead at the time of analysis. After stepwise selection, four miRNAs were included in a prognostic score. The signature was as follows: 3*hsa-miR-200c − 9*hsa-miR-29c − 2*hsa-miR-424 − 2*hsa-miR-124. With a cut-off of −52, the signature distinguished patients with good (*n* = 18) and poor (*n* = 20) prognosis with respective median survival of 47.3 months (95% CI 29.8–52.4) and 15.5 months (95% CI 9.1–22.8) (*p* < 0.001; hazard ratio 21.1, 95% CI 4.7–94.9). The same signature discriminated patients with good progression-free survival (median 19.8 months; 95% CI 15.3–33.8) from the others (median 9.1 months; 95% CI 6.3–15.5) (*p* < 0.001; hazard ratio 3.8, 95% CI 1.7–8.7).

#### Validation group

miRNAs analyses were performed on 22 patients included in the validation set (Table 2). The difference between responders and non-responders, in mean expression of the two miRNAs from the predictive signature, was not statistically significant (miR-149, *p* = 0.07 and miR-375, *p* = 0.11) (Table 9). Sensitivity, specificity, positive, and negative predictive values of the signature were 33% (3/9), 77% (10/13), 50% (3/6), and 63% (10/16), respectively. Among the four miRNAs included in the prognostic signature, only miR424 retained a borderline statistical significance for survival (HR 0.66, 95% CI 0.44–1.00, *p* = 0.05). The respective HR value for the three other miRNAs were for miR200c 0.95 (95% CI 0.53–1.70, *p* = 0.85), for miR29c 1.00 (95% CI 0.43–2.34, *p* = 0.99) and for miR124a 0.86 (95% CI 0.48–1.52, *p* = 0.59). The 4-miR signature did not anymore distinguish patients with poor and good prognosis.

**Table 9:** **Comparison between responders and non-responders of the expression of the two miRNAs contained in the predictive signature, in the derivation and the validation sets**.

	Derivation set (*N* = 38)		*P*-value (Wilcoxon)
	No response (*N* = 21)	Response (*N* = 16)	
**miR375**
Mean ± STD	5.2 ± 2.2	7.1 ± 1.8	0.01
Median (min–max)	5.8 (−0.5–7.9)	7.3 (4.2–9.8)	
**miR149**
Mean ± STD	7.2 ± 1.4	4.8 ± 1.8	<0.001
Median (min–max)	6.5 (5.0–9.5)	4.8 (1.8–7.8)	

	**Validation set (*N* = 22)**		***P*-value (Wilcoxon)**
	**No response (*N* = 13)**	**Response (*N* = 9)**	

**miR375**
Mean ± STD	3.8 ± 1.7	2.3 ± 2.3	0.07
Median (min–max)	2.8 (2.0–6.6)	1.7 (−0.2–7.6)	
**miR149**
Mean ± STD	5.4 ± 1.7	4.2 ± 1.2	0.11
Median (min–max)	5.3 (1.8–7.2)	4.3 (2.4–5.8)	

The mean and median expression of the miRNAs are expressed in ΔCT values. STD, standard deviation

We further looked at expression levels of the six selected miRNAs involved in the predictive and prognostic signatures. Statistically significant differences in the respective ΔCT values were observed when comparing derivation and validation sets (Table 10).

**Table 10:** **miRNA signatures for prediction of response to chemotherapy and of survival (validation set): differential expression according to the ΔCT values between the derivation and validation sets**.

	Derivation set (*N* = 38)	Validation set (*N* = 22)	*p* (Wilcoxon)
**miRNAs FOR RESPONSE**			
**miR375**
Mean ± STD	6.1 ± 2.2	3.2 ± 2.0	<0.001
Median (min–max)	6.2 (−0.5–9.8)	2.7 (−0.2–7.6)	
**miR149**
Mean ± STD	6.0 ± 2.0	4.9 ± 1.6	0.03
Median (min–max)	6.2 (1.8–9.5)	5.2 (1.8–7.2)	
**miRNAs FOR OVERALL SURVIVAL**			
**miR200c**
Mean ± STD	1.1 ± 2.7	−0.7 ± 1.0	<0.001
Median (min–max)	0.6 (−1.1–16.1)	−0.9 (−2.7–1.7)	
**miR29c**
Mean ± STD	5.5 ± 1.1	1.5 ± 0.7	<0.001
Median (min–max)	5.6 (2.5–7.0)	1.4 (0.2–3.0)	
**miR424**
Mean ± STD	13.7 ± 2.6	6.3 ± 1.5	<0.001
Median (min–max)	13.5 (8.6–18.3)	6.1 (4.3–9.7)	
**miR124a**
Mean ± STD	16.0 ± 3.0	7.4 ± 1.4	<0.001
Median (min–max)	18.0 (9.3–18.8)	7.8 (4.0–9.4)	

*The mean and median expression of the miRNAs are expressed in ΔCT values. STD, standard deviation*.

## Discussion

Herein, we report mature data of a prospective translational academic study performed in advanced and metastatic NSCLC, homogeneously treated with cisplatin–vinorelbine. In an attempt of searching for biological markers predicting chemosensitivity and prognostic for survival, we were able, in a derivation group, to build predictive and prognostic biological signatures based on mRNA and miRNA analyses by high throughput techniques. These signatures could not be confirmed in an independent validation group of patients treated in the same way. This study is underlying the difficulties in obtaining, in routine practice, adequate biological samples for translational research as well as the limitations for using those signatures derived from high throughput techniques.

Only 10–15% of newly diagnosed NSCLC patients will ultimately be cured (1). For the others, chemotherapy will be given at least once during the course of the disease. Cisplatin-based regimens are the cornerstone of 1st line chemotherapy for stage IV NSCLC as well as the most common combination for adjuvant therapy after surgery in completely resected stages II–III disease and for combined chemoradiotherapy. However, at least in patients treated with palliative intent, response rates are limited to 20–40% while substantial acute and chronic toxicities are encountered. A better selection of patients able to benefit, or not, from chemotherapy could theoretically help in selecting the most effective drug combinations for a particular patient; this is the purpose of customized chemotherapy. Data from randomized trials and series are suggesting that patients with poor performance status are less likely to benefit from chemotherapy than those with a good one (13). Histology is also of importance with adenocarcinomas being more susceptible to pemetrexed than squamous cell carcinomas (14). Nevertheless, there are no reliable individual predictors of chemotherapy effectiveness.

During the last decades, a better understanding of the tumors’ biology allowed in some situations to propose very active treatments when cancer is expressing a specific target. The best examples are oral tyrosine kinase inhibitors for EGFR activating mutations (5) and ALK translocation (6), with response rates around 60%, whatever the line of treatment. Other targeted therapies are currently under consideration but only a limited number of patients will have access to these very effective drugs. For cytotoxic chemotherapy, therapeutic strategies based on a genotypic analysis have been tested in randomized trials and failed to improve the common “same chemotherapy for all” approach (7, 8). Nonetheless, in these two trials, the number of considered genes was limited to ERCC1 or both ERCC1 and RRM1 that seem unlikely representing an extensive view of the biological tumor heterogeneous behavior. The problems in assessing multiple biological markers are related to the sample volume, generally limited to small biopsies in advanced and metastatic diseases, and the time necessary for performing multiple single analyses. This is an advantage for high throughput techniques like microarrays allowing yet on small samples to perform thousands of analyses at the same time. For this reason, we designed this prospective study searching for biological signatures both at the mRNA and miRNA levels.

Biological signatures have previously been published. Based on mRNA analyses, they all are dealing with survival, eventually disease-free survival, mostly in surgical stages but none was designed for searching a predictive marker of chemosensitivity. Also surgical cases are mainly representing the population used for miRNA analyses in which two signatures were tested for chemosensitivity (15, 16).

To date, more than 40 different prognostic signatures for survival, based on mRNA analyses, have been published. The signature that we identified in the derivation group was never published before. This is reflecting the large heterogeneity in the literature. In a review, only 5 genes were overlapping in a total of 327 included in seven signatures, and yet the 5 were not in common into all of them (17). Among published signatures, in most of them, there has never been any attempt to validate them in independent sets at the difference of our study. But when authors tried to do so, they cannot systematically succeed to validate their prognostic signatures (3). Potential explanations are related to heterogeneity in the patients’ and treatment’s characteristics that are frequently poorly or partially reported, to differences in the component of squamous cell and adenocarcinomas, to the statistical tests used and to the limited number of patients included in the trials that probably needs to be extensively increased for this type of translational research (18). A last component associated with a lack of reproducibility of those signatures is the heterogeneity in the platforms whatever considering the type and the number of genes tested. In a comparison of two common platforms, Affymetrix and Illumina, authors showed that the number of selected genes and their nature were not comparable with the two methods while performed on the same samples (19).

The same observations are valid for miRNA signatures. The main sources of heterogeneity are as follow: type of samples, mainly providing from surgery in patients not receiving chemotherapy but in a few cases serum has been used; the number of miRNA involved in the analyses, from a few ones to up to 880; the different techniques, single RT-PCR for very limited number of miRNA to various platforms for high throughput techniques that also are not comparable. Among more than 25 studies assessing the prognostic role of miRNA in NSCLC, the number of studies validated in an independent group of patients and taking into account other prognostic factors in a multivariate analysis was yet more limited (20–23). We found two publications analyzing the predictive role of miRNA for response to chemotherapy. In the first one, a secondary analysis from the adjuvant IALT trial including 639 completely resected NSCLC (16), 7 miRNA were considered for the analysis and the authors did not find any interaction with chemosensitivity. The second was done in a limited group of 34 SCLC patients. A three miRNA signature was associated with objective response to chemotherapy (15). We discussed the discrepancies between our study and the others in a previous publication (9).

The present study is confirming these evidences from the literature. We were able to derive, from a small but homogeneous group of patients, a single biological signature based on mRNA expression predictive of response to the cisplatin–vinorelbine regimen. We internally validated the results obtained with the microarray experiment by RT-qPCR, confirming the tumoral expression associated with the selected mRNAs and their distinction between responders and non-responders. This approach would have given us the possibility to develop a rapid, cheaper test accessible to most pathological laboratories at the difference of expensive and difficult microarrays. However, the final data from the study after attempting a validation in an independent group of patients with the same high throughput techniques was a failure despite the robust methodology of our prospective study. A major point to be primarily considered is that statistics cannot have enough power and reliability for approaching very complex biological analyses involving thousands of genes/proteins. This problem has yet been evoked in the literature when the same group using different statistics on the same samples found two distinct biological prognostic signatures (24, 25).

Our data have main strengths of which the first is the prospective nature of the present study while the majority of the published literature is dealing with retrospective studies using archival tissues. All consecutive patients presenting with suspected lung cancer were proposed to participate. This is limiting the risk of recruitment bias. As this is a prospective study, we avoided the risk of lack of data as regularly reported in retrospective series. Further, our design has foreseen, from the outset, a validation of our data in a distinct group of patients managed and treated in the same way, allowing obtaining a very homogeneous group. The validation group was treated in the same way as the derivation one and presented with similar clinical characteristics able to influence response rate and/or survival as gender, age, performance status, or stage. This was reflected in the similar response rates and survival times with no statistically significant differences (respective *p*-values of 0.86 and 0.63). The only non-statistically significant difference was linked to histology with a predominance of squamous cell carcinomas in the first group and adenocarcinomas in the second. It was previously pointed out that signatures derived in one histological type may not be applied in another, at least for survival (26, 27). Also, this is not as common to find this type of validation in the previous reported publications. Another strength of our study is underlined by the fact that we repeated the microarray experiment with the same methodology, after the failure of validation using RT-qPCR. Those microarrays as well as the RT-qPCR were commercial tests that can easily be reproducible at the difference of “home-made” techniques. Overall, we could conclude that biological signatures have to be considered for predictive or prognostic purpose only if they are externally validated in a group of patients presenting with the same clinical characteristics, managed and treated in the same ways as in the derivation group.

There are some limitations to our study. The first is the statistical design as there were no data or guidelines allowing us to calculate the adequate number of patients to be recruited. The aim of the study was finding a very efficient test able with a high probability predicting response or non-response to cytotoxic chemotherapy. For this purpose, we proposed different scenarios for which a maximum of 50 patients in the derivation group appears sufficient. Yet if this approach could be unsatisfying, other trials are generally not presenting statistical considerations on the sample size. Previous publications have underlined the risk of misclassifications according to the training-set sizes (28) and probably thousands of samples (18) would be needed to construct list of genes with strong statistical validity and reproducible results. This seems unrealistic when considering the major costs of microarrays and the difficulties in recruiting prospectively patients and samples in translational research in lung cancer. This study can only be achieved with an impressive grant from the first Belgian Cancer Plan. Further, to achieve recruitment of enough patients for transcriptomic analyses, we had to register 300 patients during a 4-year period of whom only 20% can be assessed both for response and transcriptomic analyses, most of them because no adequate tumor sample can be obtained during bronchoscopy. When discussing about the number of patients to be recruited in such trials (18), this will be a major limiting factor for further studies.

Another problem is the absence of distinction in our analyses between the tumor and its microenvironment. This could be a factor of discrepancy between the derivation and validation sets that cannot be approached by the case-mix evaluation using conventional clinical variables. According to the technique we used, the whole biopsy was directly lysed in Tripure, including both these components. Microdissection could be an option to avoid this problematic that cannot be done in the present setting at risk of degrading RNA by rapid activation of cell RNAses in those very small biopsies. We previously used successfully this technique of extemporaneous treatment of the biopsy that we were able to reproduce in the present study (29). We can further discuss the interest in the content of the signature. For predictive or prognostic purposes, it may be possible that the genes, by themselves, contained in the signatures have limited importance (30), while this is not the case if the research is dealing with assessment of biological cancer behavior, provided that the signature is reproducible in any situation. We limited the risk of additional contamination by other tissues by restricting the eligible biopsies to tumors accessible by conventional bronchoscopy, avoiding contamination by blood, skin, or muscle in case of percutaneous lung biopsy or inclusion of other tissues depending on metastatic sites (liver, nodes …). For the latter, at the time we designed the study, we did not have data regarding bio-equivalence between primary tumor and metastases, another reason why biopsies from metastatic sites were not considered.

A third limitation is related to the reference genes used for RT-qPCR normalization. In the second set, we decided using two genes, HPRT (as in the derivation set) and β-actin, as we observed for HPRT differential expression between responders and non-responders. This was ultimately the case with β-actin. We confirmed that without normalization for these reference genes, the ones contained in the signatures were not differentially expressed in the validation set (data not shown). Other authors have yet published on the limited sensitivity of various reference genes in lung cancer samples, due to nucleic acid degradation and that “stably expressed reference genes for normalization of gene expression data using RT-qPCR have not been identified” (31).

Based on the literature data and the results of our study, high throughput techniques have probably a limited usefulness for clinical application. According to the efficiency of targeted therapies, we have to evaluate if specific genetic abnormalities could have potential predictive or prognostic implications as suggested by meta-analyses. The evolution of sequencing allowing multiple evaluations of specific targets on the same sample is opening new options for future translational research.

This academic prospective translational study looking at the interest of biological signatures for predicting chemosensitivity and for prognostic purpose in advanced and metastatic NSCLC did not reach its primary objective of validating biological signatures either at mRNA or miRNA levels. According to the available literature, the role of high throughput techniques remains questionable due to their lack of reproducibility. Signatures derived from these analyses should not be anymore considered in absence of external validation in independent groups of patients with identical clinical characteristics and treatments.

## Author Contributions

All the authors gave substantial contributions to the conception/design of the study, data acquisition, and interpretation. They all revised the manuscript and approved the final version. They agree to be accountable for all aspects of the work in ensuring that questions related to the accuracy or integrity of any part of the work are appropriately investigated and resolved.

## Conflict of Interest Statement

The research was conducted in the absence of any commercial or financial relationships that could be considered as a potential conflict of interest. The study was sponsored by the Belgium National cancer Plan and the FNRS (National Scientific Research fund)-Télévie that have no influence on the design and conduct of the study, nor in the data acquisition and interpretation as well as in the writing of the manuscript.
